# Hydroponic lettuce defective leaves identification based on improved YOLOv5s

**DOI:** 10.3389/fpls.2023.1242337

**Published:** 2023-10-26

**Authors:** Xin Jin, Haowei Jiao, Chao Zhang, Mingyong Li, Bo Zhao, Guowei Liu, Jiangtao Ji

**Affiliations:** ^1^ College of Agricultural Equipment Engineering, Henan University of Science and Technology, Luoyang, China; ^2^ Science and Technology Innovation Center for Completed Set Equipment, Longmen Laboratory, Luoyang, China; ^3^ State Key Laboratory of Soil - Plant - Machine System Technology, Chinese Academy of Agricultural Mechanization Sciences, Beijing, China; ^4^ Eponic Agriculture Co., Ltd, Zhuhai, China

**Keywords:** defect detection, EBG_YOLOv5, ECA, BiFPN, GSConv

## Abstract

Achieving intelligent detection of defective leaves of hydroponic lettuce after harvesting is of great significance for ensuring the quality and value of hydroponic lettuce. In order to improve the detection accuracy and efficiency of hydroponic lettuce defective leaves, firstly, an image acquisition system is designed and used to complete image acquisition for defective leaves of hydroponic lettuce. Secondly, this study proposed EBG_YOLOv5 model which optimized the YOLOv5 model by integrating the attention mechanism ECA in the backbone and introducing bidirectional feature pyramid and GSConv modules in the neck. Finally, the performance of the improved model was verified by ablation experiments and comparison experiments. The experimental results proved that, the Precision, Recall rate and mAP_0.5_ of the EBG_YOLOv5 were 0.1%, 2.0% and 2.6% higher than those of YOLOv5s, respectively, while the model size, GFLOPs and Parameters are reduced by 15.3%, 18.9% and 16.3%. Meanwhile, the accuracy and model size of EBG_YOLOv5 were higher and smaller compared with other detection algorithms. This indicates that the EBG_YOLOv5 being applied to hydroponic lettuce defective leaves detection can achieve better performance. It can provide technical support for the subsequent research of lettuce intelligent nondestructive classification equipment.

## 1 Introduction

Hydroponic lettuce not only has a large market demand, but also has a short growth cycle (about 45d) with high economic value, therefore, it has become one of the most widely grown vegetables on indoor farms. However, the leaves of hydroponic lettuce are dense and delicate, which will be easily damaged to a certain extent during the harvesting process. And after harvesting, lettuce leaves will easy to appear yellowing, wilting even decay. Especially when the leaves decayed, it will not only affect appearance but also infects other good quality leaves, and even the nitrite content will sharply increase (Yan et al., 2015; Van Gerrewey et al., 2021). These defective leaves will shorten the shelf life of lettuce and also can produce a certain degree of commodity value loss. Currently, a visual judgment is the primary method used by human to identify defective leaves of hydroponic lettuce. This method is time-consuming and laborious, and will be affected by human subjective factors. Therefore, it is of great significance to realize intelligent detection of defective leaves of hydroponic lettuce.

In recent years, traditional machine vision technology has been widely used in the field of agricultural defect detection (Dang et al., 2020; Zhang et al., 2021). Sun et al. (2012) used the mixed fuzzy cluster separation algorithm (MFICSC) to achieve the target clustering segmentation of lettuce image, which provided a reference for the non-destructive detection of lettuce physiological information. Kong et al. (2015) used the feature parameters extracted from the lettuce image for three-dimensional visualization modeling, and intuitively reflected the growth state of the lettuce through the visualization. Hu et al. (2014) designed k-means algorithm to detect the appearance defects of bananas, the initial step in k-means was utilized to categorize the foreground and background of bananas, and the second step of k-means was employed to quantify the damage lesions on the surface of bananas. Li et al. (2002) developed a computer vision-based system for detecting surface defects on apples. The system normalizes the original image, subtracts it from the original image, and extracts defective parts of the apple surface through threshold segmentation. Kumar et al (Prem Kumar and Parkavi, 2019). utilized machine vision technology for the purposes of detecting and evaluating the quality of fruits and vegetables, which solved the problem of slow manual efficiency. The above methods are all based on traditional machine vision methods for image preprocessing and feature extraction. However, crops have different defect characteristics, and manual selection of feature variables results in limitations in the promotion and application of these methods.

With the development of machine learning, deep learning has been widely applied in agricultural product defect detection. Muneer et al (Akbar et al., 2022; Hussain et al., 2022). proposed a new lightweight network (Wlnet) based on VGG-19 network for the detection of peach leaf bacteria. The WLnet model was trained with self-built peach leaf bacteria dataset, and the experimental results showed that the recognition accuracy reached 99%. Alshammari et al. (2023) proposed an optimized artificial neural network to identify olive leaf diseases. Whale Optimization Algorithm was used to select necessary features, and finally, artificial neural network was used to classify the data. The experimental results showed that this model is superior to the existing model in terms of accuracy and recall rate. Li et al. (2021) employed the enhanced Faster-RCNN (Ren et al., 2015) architecture to identify the growth status of hydroponic lettuce seedlings, with an average accuracy rate of 94.3% and 78.0% for dead seedlings and double-plant seedlings, respectively. Compared with SSD (Liu et al., 2016) and Fast R-CNN, YOLO (Redmon et al., 2016; Redmon and Farhadi, 2017; Redmon and Farhadi, 2018; Bochkovskiy et al., 2020) networks are more concise, accurate and effective, making them widely used in agricultural defect detection. Liu et al (Liu and Wang, 2020). used an image pyramid method to optimize the feature layer of the YOLOv3 model, achieving efficient multi-scale feature detection. The detection accuracy of this algorithm is 92.39% and the detection time is 20.39 ms. Wang et al (Wang and Liu, 2021). improved YOLOv4 by adding a dense connection module, and the average accuracy and detection time of tomato disease identification reached 96.41% and 20.28ms, respectively. Yao et al. (2021) proposed a Kiwifruit defect detection model based on improved YOLOv5. The experimental results show that the mAP_0.5_ of this model is 94.7%. Abbasi et al. (2023) proposed an automatic crop diagnosis system for detecting diseases in four hydroponic vegetables: lettuce, basil, spinach, and parsley. This study selected YOLOv5s as the detection model with mAP_0.5_ 82.13% and detection speed of 52.8 FPS. Hu et al. (2022) proposed a method for identifying cabbage pests based on near-infrared imaging technology and YOLOv5. The experimental results showed that mAP reaches 99.7%.

There are many research on the detection of spherical fruit defects, but there is generally little research on the detection of defective leaves of hydroponic lettuce. The detection and location of defective leaves of hydroponic lettuce by deep learning can provide a new solution for intelligent non-destructive detection of hydroponic lettuce quality. Therefore, this study aims to propose a method for detection of defective leaves of hydroponic lettuce based on improved YOLOv5, namely EBG_YOLOv5(E-ECA, B-BiFPN, G-GSConv). First, an image acquisition system was designed and used to obtain images of defective leaves of hydroponic lettuce. Secondly, introducing the ECA module into the backbone of the YOLOv5 model to improve the learning ability of the model for the features. The BiFPN and GSConv module was introduced into the Neck of the YOLOv5 model to improve feature fusion and accuracy of the model. Finally, the EBG_YOLOv5 model performance was verified by ablation and comparison experiments.

## 2 Materials and methods

### 2.1 Image sample acquisition

**Figure 1:**
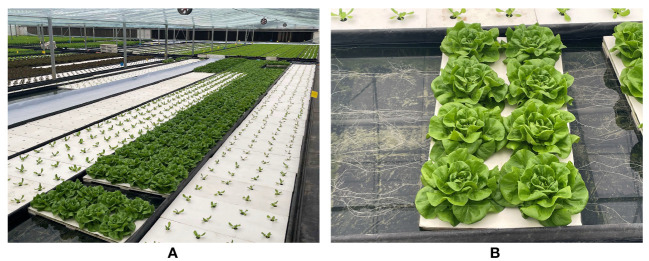
Lettuce greenhouse: **(A)** Lettuce cultivation environment; **(B)** Lettuce growth status.

The hydroponic lettuce sample used in this study is cream lettuce Huisheng No. 1 from Ensheng Hydroponic Vegetable Base, Li Lou Town, Luoyang City, Henan Province, China. The growth environment temperature of lettuce is 15 to 25 °C, the humidity is controlled at 60 to 75%, and the growth period is 25 to 30 days. The cultivation environment and growth status of lettuce are shown in **Figure 1**. After the lettuce is ripe, it is manually harvested and then photographed in the indoor greenhouse and laboratory from April to May 2023.

**Figure 2:**
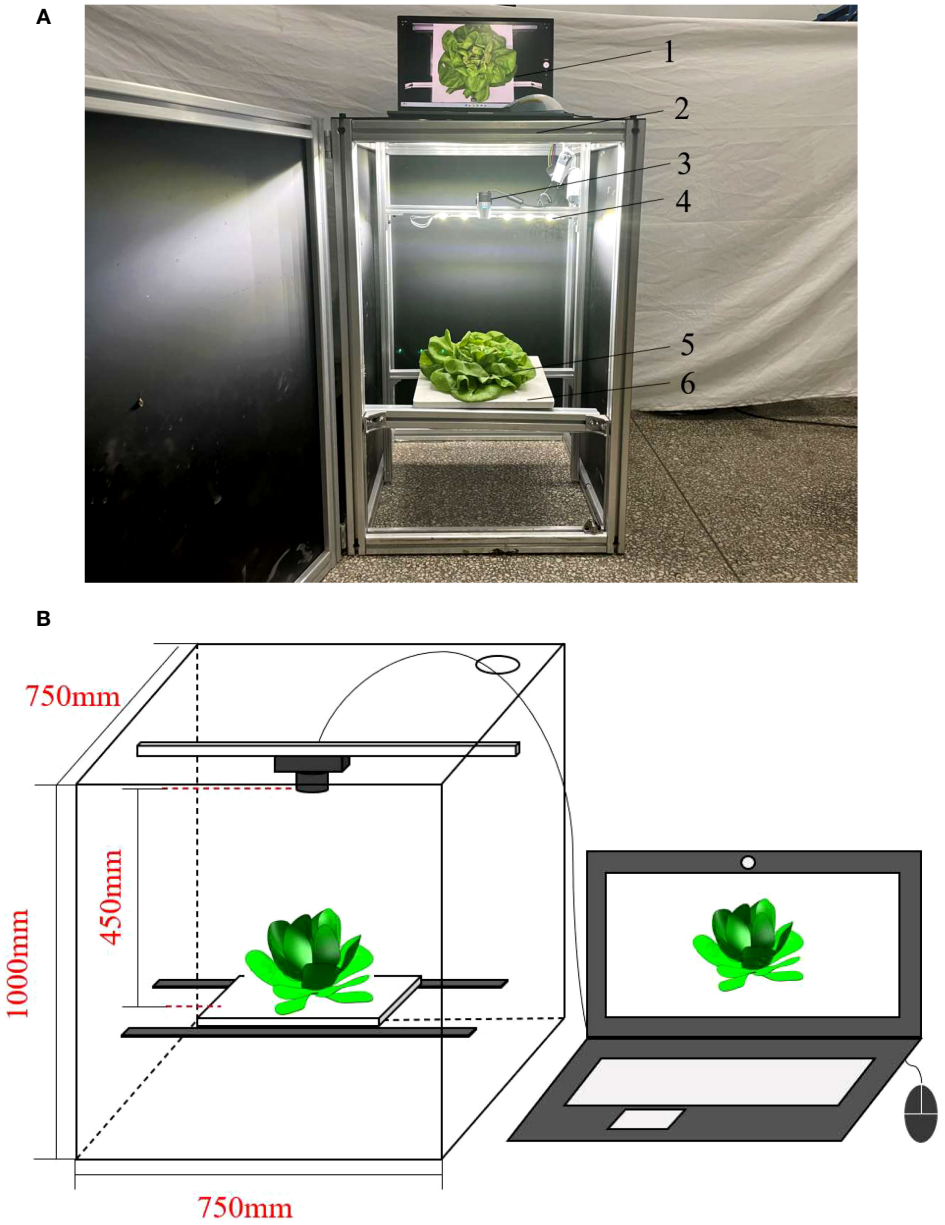
Image acquisition apparatus: **(A)** Physical drawing of image acquisition apparatus; **(B)** Schematic diagram of image acquisition apparatus; 1. Computer, 2. Obscura, 3. Camera, 4. Light source, 5. Cream lettuce, 6. carrier plate.

In the laboratory, an image acquisition system was built, which consists of a camera, a camera obscura, a carrier plate and an illumination source, as shown in **Figure 2**. The camera model is Microsoft Lifecam Elite Edition (Redmond, USA), and the image resolution is 1920 × 1080 pixels. To prevent the influence of a singular shooting background on network learning, images of lettuce leaves were added into the dataset. During the image acquisition process, the distance between the camera and the carrier plate was predetermined and remains constant, the distance was 450 mm. A total of 1200 pictures of lettuce defective leaves in greenhouse and laboratory environment were collected, and all images were adjusted to 640 × 270 pixels before network training.

### 2.2 Dataset construction

**Figure 3:**
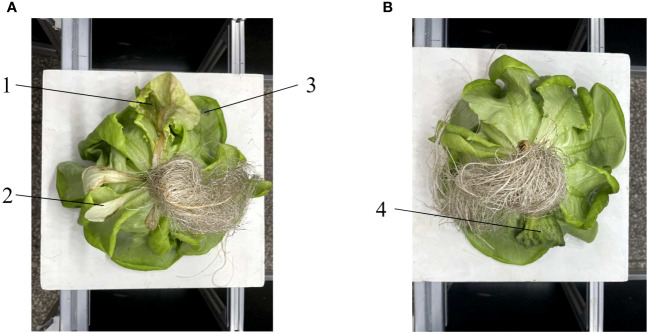
Examples of defective leaves of hydroponic lettuce: 1. Decayed, 2. Yellow, 3. Broken, 4. Wilting. **(A)** Lettuce with decayed, yellow and broken leaves. **(B)** Lettuce with Wilting leaves.

In this study, the defective leaves of hydroponic lettuce were divided into four categories: Decayed, Broken, Yellow and Wilting, the color of broken leaves is the same as that of healthy leaves, and the color of yellow leaves, wilting and decayed defects becomes yellow, dark green and black, respectively, as shown in **Figure 3**. Secondly, the leaf texture of hydroponic lettuce in different states was also different, the texture of yellow leaves did not change significantly. The wilted leaves were wrinkled due to water loss, but basically maintained the shape of the leaves. The decayed leaves became soft, the leaf texture disappeared, and there is no fixed shape; The broken leaf texture was destroyed, with obvious cracks or holes.

The defective leaves in the image were annotated by LabelImg image annotation software, with Decayed as D (No.0), Broken as B (No.1), Yellow as Y (No.3), and Wilting as W (No.4). After annotation, an xml file in VOC format is generated, which contains the image size, the coordinate position of the defective leaves, and various label names. Then, the xml file was converted into the txt file corresponding to the YOLO model. Finally, the images of lettuce and the labeles were divided into a training set and a test set in an 8:2 ratio, and placed in images and labels folders, respectively.

### 2.3 Data augmentation

Deep learning algorithm training requires a large dataset to continuously extract and learn features, but the data collection process is very time-consuming. Therefore, offline data augmentation was conducted on the original dataset before model training, aiming to increase the number and diversity of samples on the basis of limited data, and improve the robustness and generalization ability of the network model. In the experiment, the augmentation methods adopted include: translation, mirror, cropping, Gaussian noise and brightness adjustment, etc. A total of 3600 images are obtained after enhancement.

**Figure 4:**
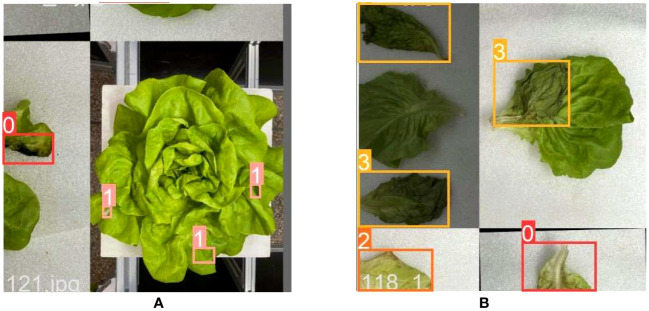
Examples of mosaic image augmentation results: **(A)** Whole lettuce image; **(B)** Single leaf image.

In addition to offline augmentation operations, the model training process also uses Mosaic data augmentation technology. Randomly read 4 images in the training set for random cropping, rotation, scaling, and other operations, and then concatenate them into one image as training data. The processing results are shown in **Figure 4**.

## 3 Hydroponic lettuce defective leaves identification network

### 3.1 YOLOv5s network model

The main architectures of YOLOv5s include Input, Backbone, Neck, and Prediction. In the input part, Mosaic data enhancement, adaptive anchor box calculation, and adaptive image scaling are used to enrich the data and improve the training speed of the network. The Conv module, C3 module, and SPPF module are the main components of the backbone network. Among them, the C3 module is primarily used for feature extraction from images, and the SPPF module pools feature maps in different dimensions to generate semantic information. The Neck part adopts FPN (Feature Pyramid Networks) and PAN (Path Aggregation Network) structure. FPN generates image semantic information in a top-down manner, while PAN supplements target location information in a bottom-up manner. The Prediction part analyzes the feature maps of different scales generated by the Neck, and provides the category probability and positioning information of the target.

### 3.2 YOLOv5s network improvements

**Figure 5:**
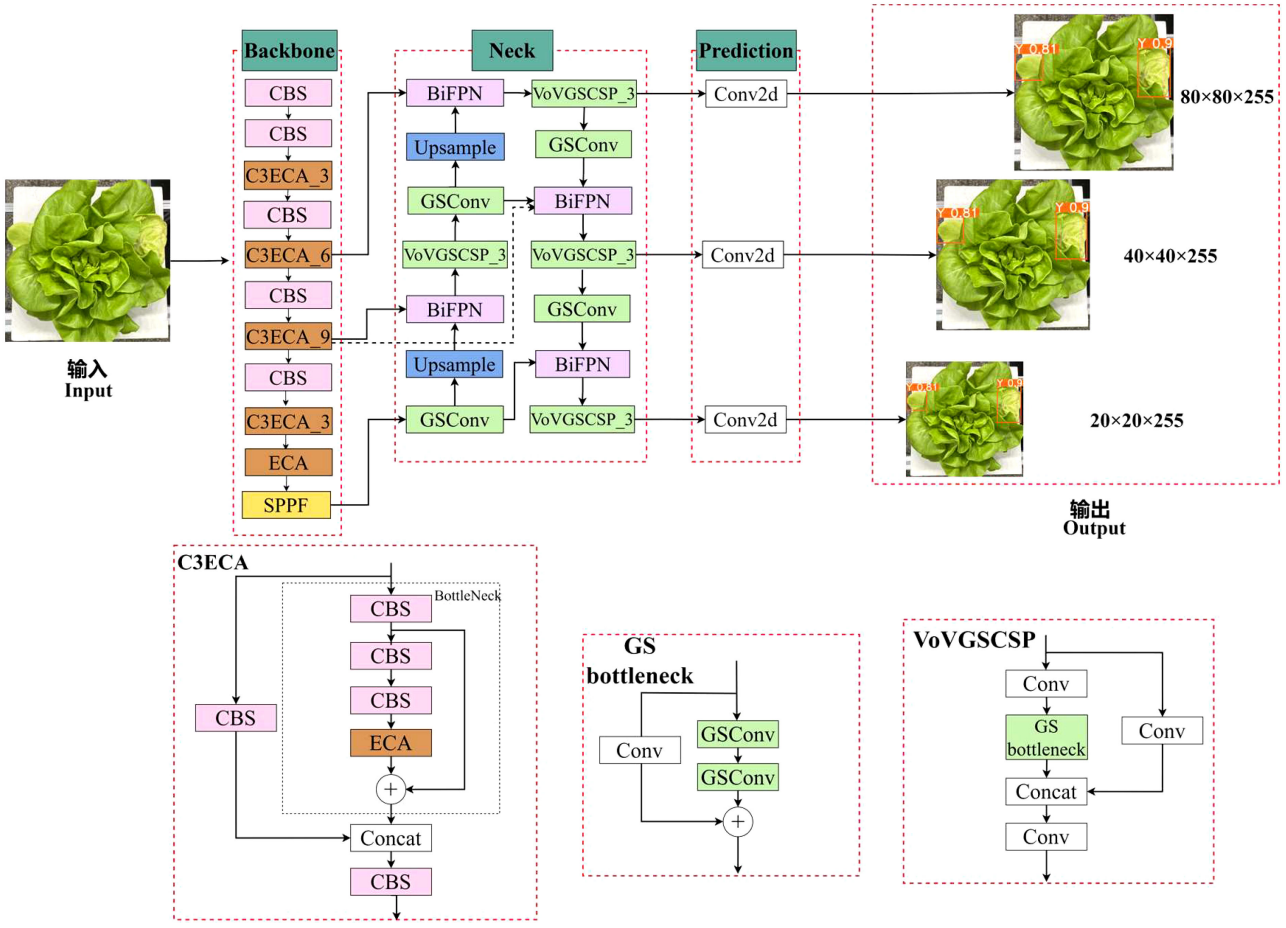
Improved YOLOv5 model: CBS is a convolution unit; the number of the C3ECA module represents its quantity. ECA is an attention module; SPPF represents spatial pyramid pooling; GSConv is a newly introduced convolution unit; upsample is feature upsampling; the number behind the VoVGSCSP module represents the quantity of the module; Concat represents feature stitching; Conv2d represents two-dimensional convolution; 80 × 80 × 255, 40 × 40 × 255, and 20 × 20 × 255 represents the length, width, and depth of different dimensions of the network output feature map.

The module responsible for extracting image features in YOLOv5s is C3 module (Concentrated Comprehensive Convolution Block). As the network deepens, the texture and contour information useful for identifying small targets gradually decreases. After being processed by several C3 modules, the positional data of occluded and small targets in the image becomes inaccurate, and the feature data is easily loss. Therefore, the model may encounter error detection and omissions when identifying small or occluded targets. The defective leaves of hydroponic lettuce vary in size, and some defective leaves may be obstructed by the roots, which cannot be accurately identified in actual testing. To enhance the detection accuracy of defective leaves of hydroponic lettuce, EBG_YOLOv5s model is proposed in this research. The particular framework was presented in **Figure 5**, the improvement are mainly reflected in the following three aspects.

(1) Introducing Efficient Channel Attention (ECA) into the C3 module of the backbone network to reconstruct the C3 module into a C3ECA module, and then add the ECA module after the last layer of C3ECA module. Attention mechanism can enhance the ability to extract image features and fully utilize limited feature information.
(2) In the neck part, the BiFPN (Bidirectional Feature Pyramid Network) structure is used to establish bidirectional cross-scale connections, which incorporate learnable weights to enhance feature fusion and improve detection accuracy.
(3) The neck adopts GSConv lightweight convolution instead of traditional convolution. In addition, the C3 module is replaced by VoV-GSCSP bottleneck module composed of GSConv modules. GSConv can reduce computational complexity while ensuring the accuracy, while VoV-GSCSP can reduce model inference time and improve accuracy.

#### 3.2.1 ECA module

**Figure 6:**
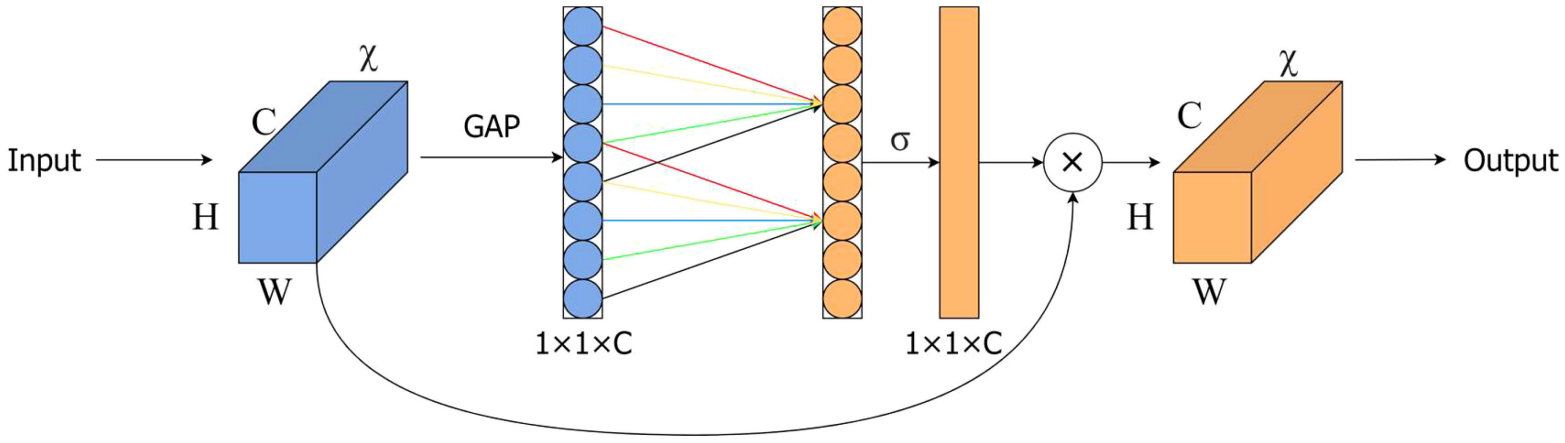
ECA (Efficient Channel Attention) module.

This study introduced an attention mechanism to the YOLOv5s network to extract feature information and enhance the identification of defective leaf characteristics. In order to keep the model lightweight, when adding attention modules, it is necessary to consider improving performance without increasing model complexity. Therefore, we introduced the ECA (Wang et al., 2020) module into the model, and its structure is shown in **Figure 6**.

**Figure 7:**
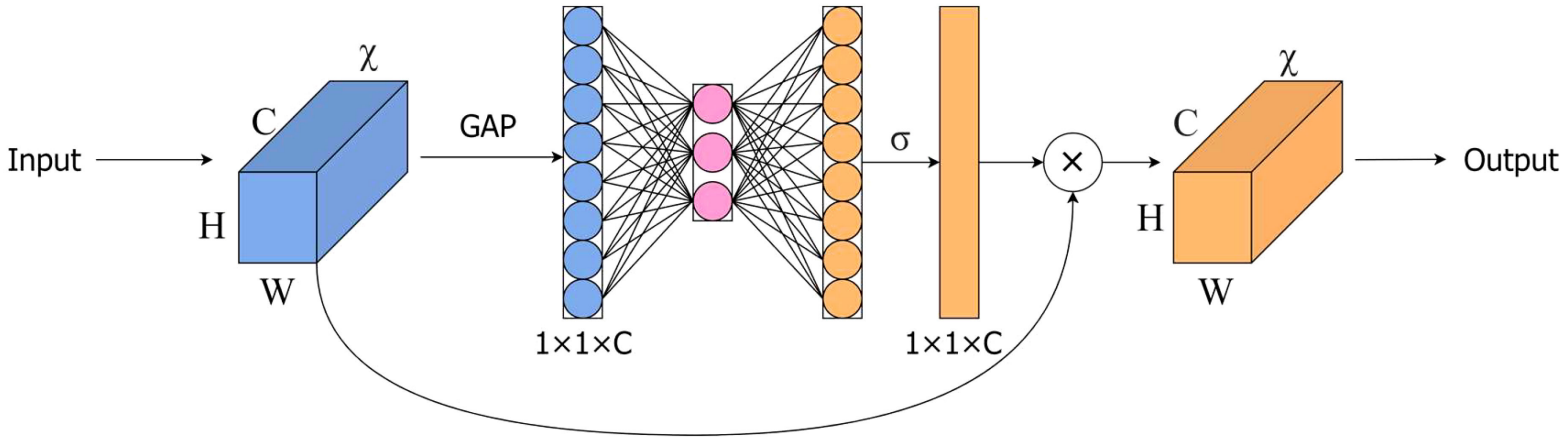
SE (Squeeze and Excitation Network) module.

The ECA module is an extremely lightweight attention module that combines channelization technology from SENet (Squeeze and Stimulation Network) (Hu et al., 2018). As shown in **Figure 7**, the SENet structure amplifies channel correlation by using two fully connected layers after global average pooling, and extracts features by reducing and then increasing dimension. However, this method performs poorly in distinguishing complex backgrounds from target features. The ECA module only uses one-dimensional convolution to capture cross-channel nonlinear information, thereby reducing computational requirements and enabling the network to learn channel information more efficiently.

In **Figure 6**, H, W and C represent the height, width, and channel dimensions of the feature map, respectively. GAP represents to the global average pooling layer, the symbol σ denotes the Sigmoid activation function, the value of k represents the size of the adaptive convolution kernel, which indicates the local cross-channel interaction coverage. The coverage of the interaction is proportional to the channel dimension C. Therefore, there is a mapping relationship between k and channel dimension C:


(1) $$\begin{array}{c} C=\phi \left(k\right) \end{array}$$

Where *ϕ* represents the optimal mapping. Considering that the quantity of channels typically increases exponentially by a factor of 2, a nonlinear model is applied to estimate the mapping function *ϕ*:


(2) $$\begin{array}{c} C=\phi \left(k\right)=2^{\left(\gamma \times k-b\right)} \end{array}$$

The expression for the coefficient k can be formulated as.


(3) $$\begin{array}{c} k=\psi \left(C\right)={\left|\frac{\log_{2}C+b}{\gamma }\right|}_{odd} \end{array}$$

Where γ and b denote the nonlinear parameters of the linear regression and |*t*|_add_ is the nearest odd integer to t:

#### 3.2.2 BiFPN module

**Figure 8:**
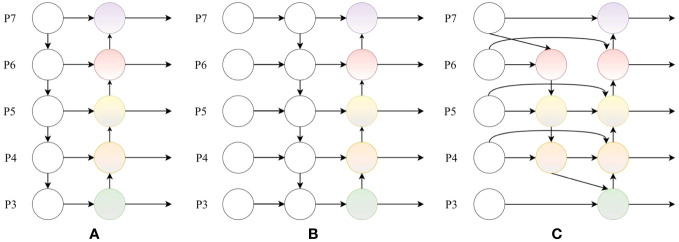
**(A)** FPN structure; **(B)** PAN structure; **(C)** BiFPN structure.

The YOLOv5s network adopts FPN and PAN pyramid modules in the neck. Both can effectively maintain the detailed features of the target. However, excessive attention to model details often leads to overfitting and reduces the model’s generalization ability. In the dataset of defective hydroponic lettuce leaves, different defect types have differences in shape, texture, color, and other aspects. In the network training process, different input features often have uneven contribution rates. Therefore, the BiFPN (Tan et al., 2020) module is used in the Neck. On the basis of the PAN structure, BiFPN transitions from a unidirectional connection to a bidirectional cross-scale connection, and achieves higher level feature fusion through repeated stacking. It introduces adjustable weights to acquire an understanding of the importance of various input features, thereby improving efficiency and accuracy. The structure of FPN, PAN, and BiFPN are shown in **Figure 8**.

#### 3.2.3 GSConv and VoVGSCSP module

For agricultural product defect detection, speed and accuracy are equally important. To enhance the precision of identifying defective leaves in hydroponic lettuce and kepp real-time detection, we introduced GSConv into the Neck part of YOLOv5s, and the C3 module was replaced by the VoVGSCSP module.

SConv (Standard Convolution) operates on three channels at the same time, the number of convolutional kernels is equal to the number of output channels, and the number of channels in convolutional kernels is equal to the number of input channels. As the network deepens, excessive use of SConv can lead to an accumulation of parameter and computational complexity. Ghostconv (Han et al., 2020) module is proposed by Han K et al., which can effectively extract image features while reducing the number of parameters, but will lose a lot of channel information in its operation.

**Figure 9:**
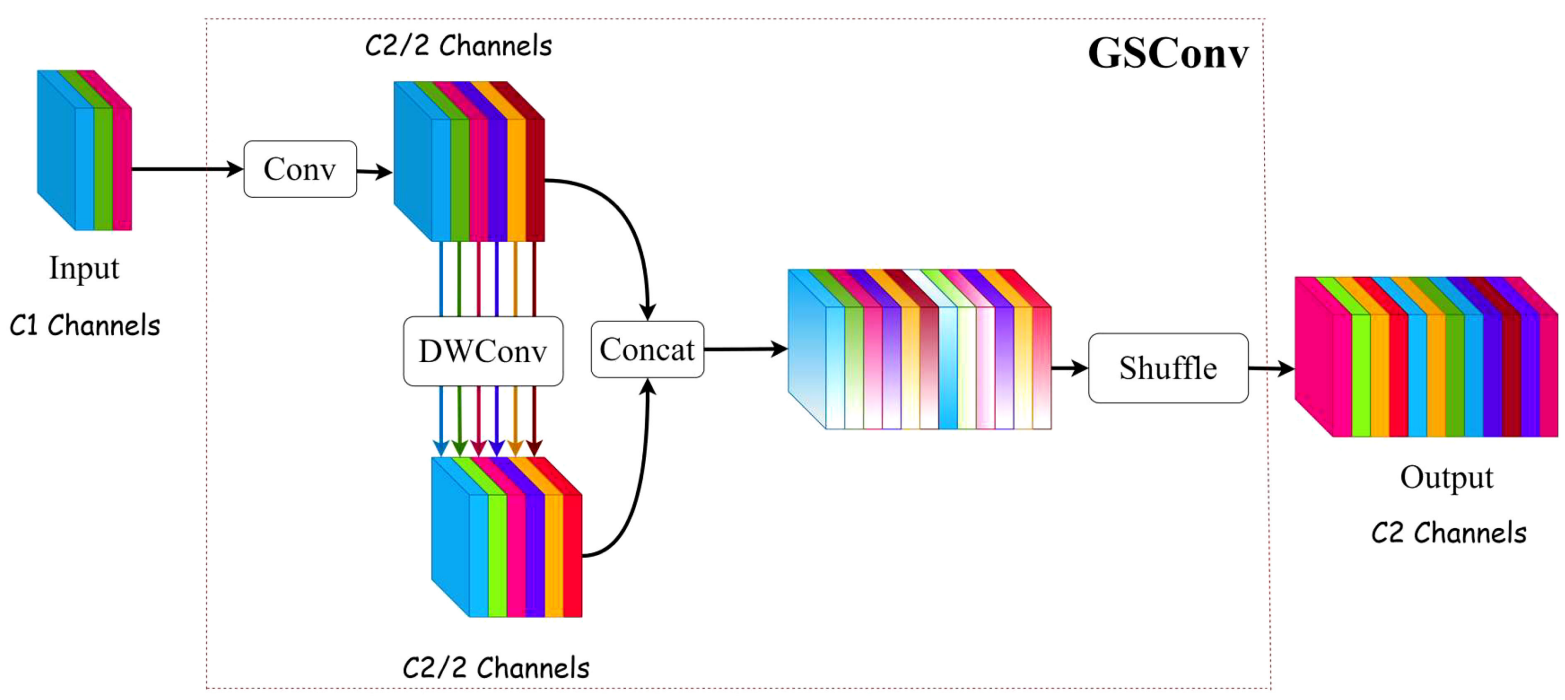
GSConv structure.

To resolve the issues pertaining to the convolution module aforementioned, Li et al. (2022) proposed a lightweight convolution module GSConv, the structure is shown in **Figure 9**. Assuming that C1 represents the number of input channels and C2 represents the number of output channels. Firstly, a standard convolution is performed, the number of channels is adjusted to half of the original number, denoted as C2/2. Secondly, a DWConv (Depthwise separable convolution) is performed, with the channel number unchanged. Finally, the results of two convolutions are concatenated and shuffled to output a result. The shuffling operation can evenly disrupt the channel information, enhance the extracted semantic information, strengthen the feature fusion and optimize the representational ability of image features.

Building upon GSConv, the GS bottleneck VoVGSCSP module adopts a one-shot aggregation technology to optimize the cross-stage network component. This method effectively reduces computation and simplifies network structure, while still achieving satisfactory accuracy.

### 3.3 Model evaluation measures

To comprehensively evaluate the performance of the model, we uesd Precision (P), Recall rate (R), mean Average Precision (mAP), model size, parameters, GFLOPs and detection speed (FPS) as evaluation indicators. The P represents the precision of the model, while the R indicates its ability to detect positive samples. mAP_0.5_ represents the average AP of all categories when the IoU threshold is set to 0.5. The larger the value, the higher the recognition accuracy of the model. The calculation formula is as follows:


(4) $$\begin{array}{c} P=\frac{TP}{TP+FP} \end{array}$$

(5) $$\begin{array}{c} R=\frac{TP}{TP+FN} \end{array}$$

(6) $$\begin{array}{c} P=\int_{0}^{1}P\left(R\right)dR \end{array}$$

(7) $$\begin{array}{c} mAP=\frac{1}{N}\sum_{i=1}^{N}AP_{i} \end{array}$$

In the above formula, TP represents the count of positive samples accurately classified as positive; FN designates the quantity of positive samples inaccurately categorized as negative; FP indicates the number of negative samples misclassified as positive; while N represents the number of classes encompassed in the dataset.

### 3.4 Experimental environment and parameter settings

The experimental environment for this study includes the Windows 10 operating system, Intel Core i7-11800H with 16GB of memory, NVIDIA GeForce RTX3060 with 8GB of memory, PyTorch deep learning framework, PyCharm development environment, CUDA 10.2.0 and cudnn 7.6.5 versions.

During the training phase, the batch size was set to 8, the weight decay was set to 0.0005. The SGD momentum was set to 0.9, and the initial learning rate is set to 0.01. The model is trained over a period of 300 epochs.

## 4 Experiment and result

### 4.1 Comparison of various attention mechanisms

**Figure 10:**
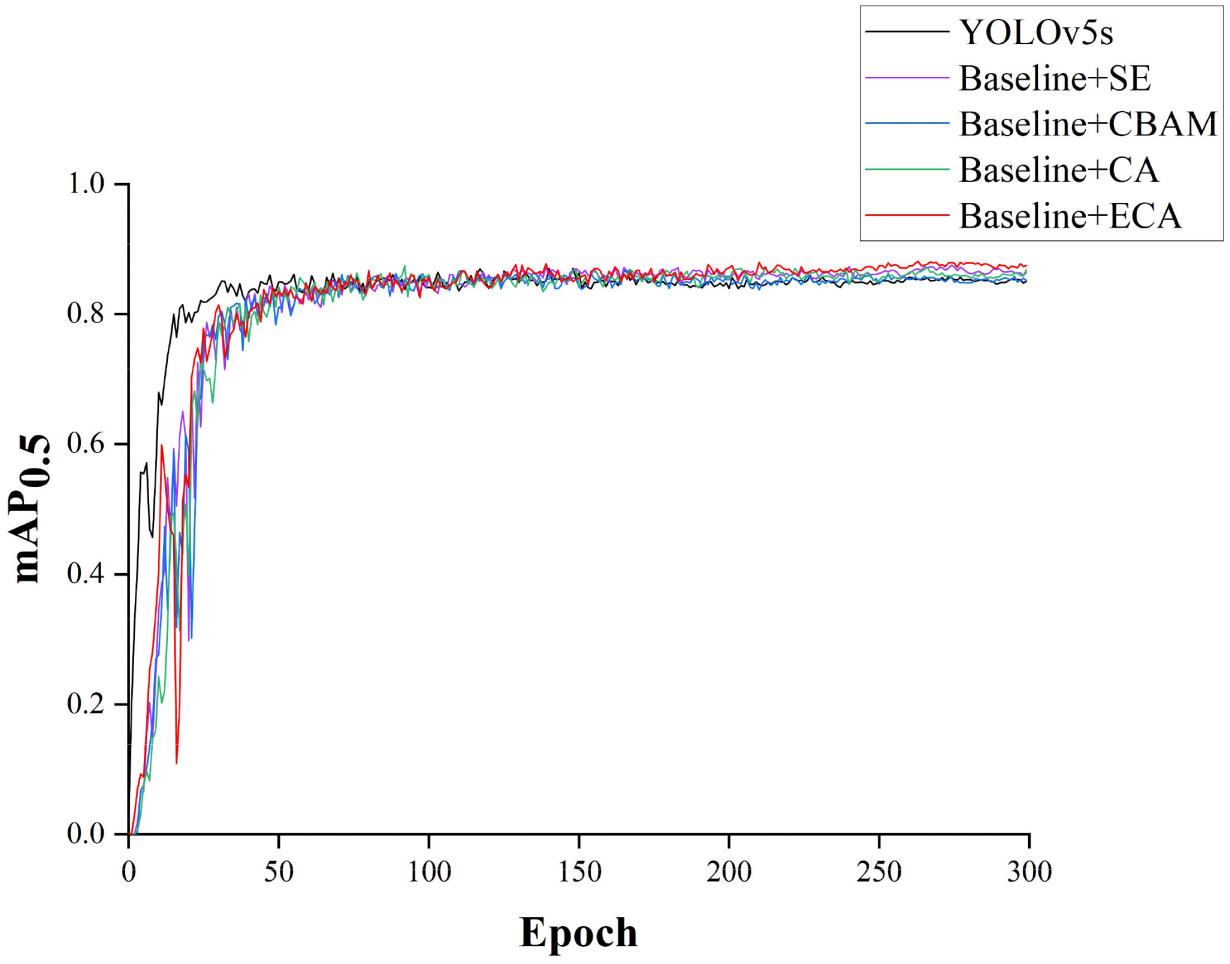
Comparison of mAP0.5 attained in fusion experiments featuring distinct attention mechanisms.

To validate the effectiveness of ECA model, the network introducing BIFPN, GSConv and VOVGSCSP modules was selected as the baseline and the model performance was compared under different attention mechanisms. Three attention modules, CBAM (Woo et al., 2018), SE and CA (Hou et al., 2021), were selected to replace ECA in the network under the same experimental environment. The results of the experiment are shown in **Table 1**, and the mAP_0.5_ comparison curve for each model are shown in **Figure 10**.

**Table 1:** Results of comparative experiments with fused attention mechanisms.

Models	Pr/%	Re/%	mAP_0.5_/%	Weights/MB	GFLPOs	Parameters
YOLOv5s	88.9	82.3	85.4	13.7	15.8	7020913
Baseline+CBAM	89.9	81.2	85.2	11.7	12.9	5925610
Baseline+SE	91.2	81.8	87.2	11.7	12.8	5924826
Baseline+CA	89.0	82.1	85.8	11.7	12.9	5923186
Baseline+ECA	89.0	84.3	88.0	11.6	12.8	5876594

From **Table 1**, it can be seen that compared with the YOLOv5s, the model with CBAM module has slight improvement in Precision, while the Recall rate and mAP_0.5_ have both decreased. The model with SE module and the model with CA module is improved Precision and mAP_0.5_, but the recall rate is reduced. After introducing the ECA module, the Precision of the model was improved by 0.1%, the recall rate was improved by 2.0%, and mAP0.5 was improved by 2.6%. In addition, compared to other modules, the models with ECA modules have the smallest model weight, computational cost, and parameters. The above results fully indicate that in the self-constructed dataset of this study, the ECA module outperforms other attention modules.

### 4.2 Ablation of experiments

In order to verify the effectiveness of the improved model, ablation experiments were performed. The experimental results are shown in **Table 2**. The E_YOLOv5 represents that C3ECA and ECA module were added to the backbone. The B_YOLOv5 represents that introducing BiFPN structure. TheG_YOLOv5 represents the introduction of GSConv and VoVGSCSP modules in Neck to replace traditional convolutional and C3 module. The EBG_YOLOv5 represents using three strategies at the same time.

**Table 2:** Ablation experimental result.

Models	ECA	BiFPN	GSConv	Pr/%	Re/%	mAP_0.5_/%	Weights/MB	GFLPOs	Parameters
YOLOv5s	**×**	**×**	**×**	88.9	82.3	85.4	13.7	15.8	7020913
E_YOLOv5	**√**	**×**	**×**	90.4	81.9	86.7	13.8	15.8	7020937
B_YOLOv5	**×**	**√**	**×**	87.4	85.7	87.0	13.8	16.0	7086458
G_YOLOv5	**×**	**×**	**√**	90.7	82.1	87.2	11.5	12.6	5843793
EB_YOLOv5	**√**	**√**	**×**	89.5	83.4	87.2	13.8	16.0	7086482
EBG_YOLOv5	**√**	**√**	**√**	89.0	84.3	88.0	11.6	12.8	5876594

“√” indicates that this method is used.

As shown in the table, when the attention module ECA is fused in YOLOv5s, the Precision and mAP0.5 of E_YOLOv5 are improved by 0.5% and 1.3%, respectively, but the recall rate decreases by 0.4%. And the model weight and parameters only increased by 0.1MB and 24, respectively, without increasing the calculation cost. This indicates that adding the ECA module improves the overall performance of the model without increasing computational cost, parameters and weight. When only replacing the feature pyramid architecture, the Recall rate and mAP0.5 increased by 3.4% and 1.6%, respectively, while the Precision decreased by 1.5%. In addition, the weight of the model increased by 0.1MB, GFLOPs increased by 0.2, and parameters increased by 65545. Therefore, the introduction of the BiFPN structure improved the ability of model to find positive examples, but the feature fusion mechanism resulted in a certain loss of precision of the model. By adding the GSConv and VoVGSCSP modules to the Neck part, the Precision and mAP0.5 increased by 0.8% and 1.8%, respectively. Meanwhile, the weights, GFLOPs, and parameters of the model decreased by 2.2MB, 3.2, and 1177120, respectively. This shows that GSConv can more fully learn the features of lettuce leaf and has lower computational costs compared to standard convolutions.

By introducing ECA and BiFPN modules into YOLOv5, the Precision, Recall rate, and mAP0.5 were improved by 0.6%, 1.1%, and 1.8%, respectively, compared to YOLOv5. Compared to E_YOLOv5 and B_YOLOv5, EB_YOLOv5 compensates for the shortcomings in Precision or Recall rate of single module, indicating the combination of ECA and BiFPN to enhance model performance. With three strategies used in YOLOv5, compared to YOLOv5s, the Precision, Recall rate, and mAP0.5 of EBG_YOLOv5 increased by 0.1%, 2.0%, and 2.6%, respectively. Meanwhile, the model weights, GFLOPs, and parameters were reduced by 2.1MB, 3.0, and 1144319, respectively. Therefore, the above results can fully illustrate the effect of this paper on the model improvement.

**Figure 11:**
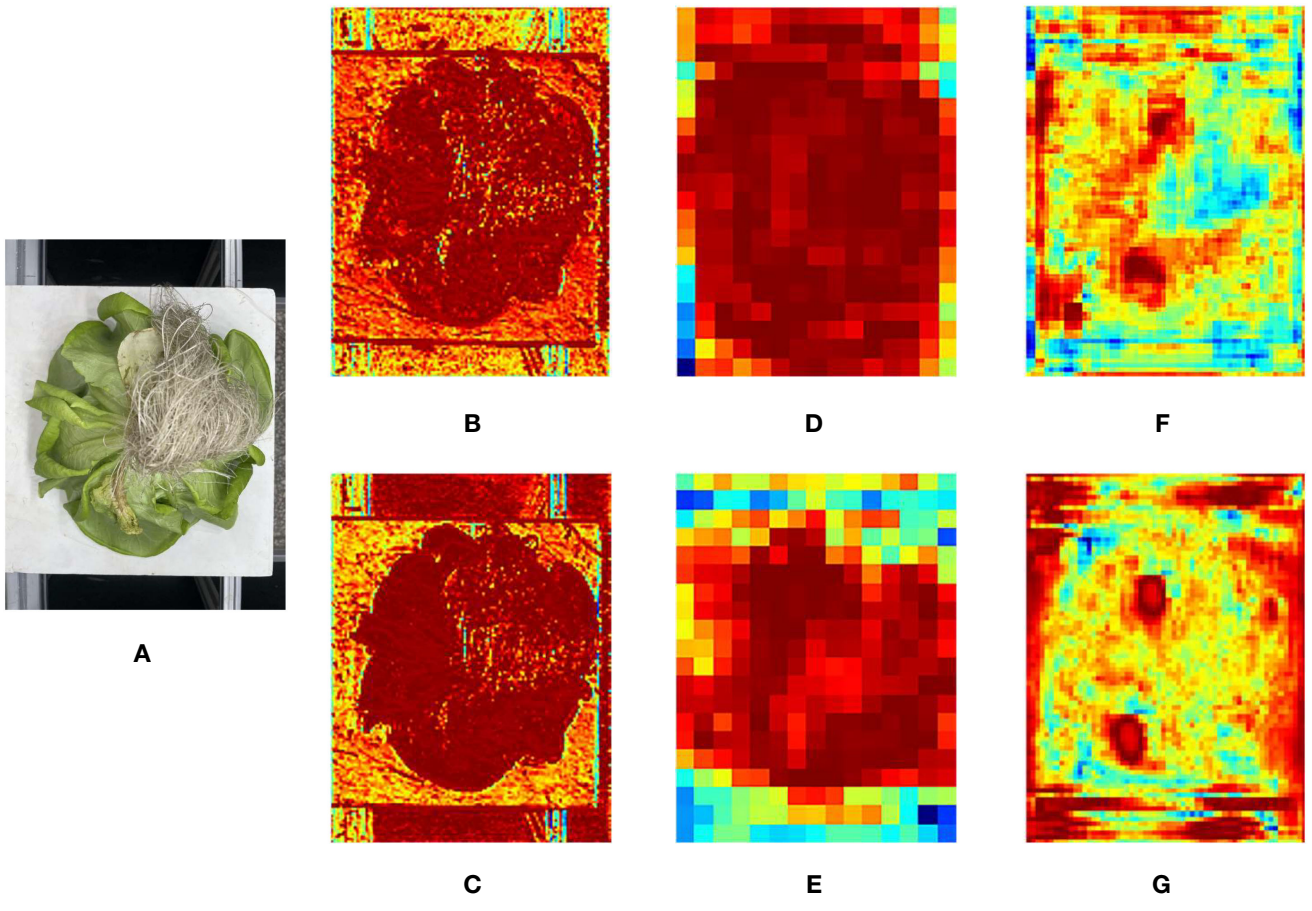
Original and intermediate feature map of Hydroponic lettuce: **(A)** Original picture; **(B)** YOLOv5s C3 (2th layer) output; **(C)** E_YOLOv5 C3ECA (2th layer) output; **(D)** YOLOv5s Concat (12th layer) output; **(E)** B_YOLOv5 Bifpn (12th layer) output; **(F)** YOLOv5s C3(17th layer) output; **(G)** G_YOLOv5 VoVGSCSP (17th layer) output.

Based on ablation experiments, in order to reflect the influence of each improvement strategy on feature extraction, this study visualized the results using heat feature maps and analyzed and compared them, as shown in **Figure 11**. **Figure 1A** shows the original image, **Figure 11B** shows the feature map generated by the C3 module (2th layer) of the YOLOv5s, and **Figure 11C** shows the feature map generated by the C3ECA module (2th layer) of the E_YOLOv5s. **Figure 11D** shows the feature map outputted by the Concat module (12th layer) of the YOLOv5s, and **Figure 11E** shows the feature map outputted by the BiFPN module (12th layer) of B_YOLOv5. **Figure 11F** shows the feature map outputted by the C3 module (17th layer) of the YOLOv5s, while **Figure 11G** shows the VoVGSCSP module (17th layer) outputted of G_YOLOv5.

From the thermal feature map, it can be seen that after the introduction of the ECA module, the model pays more attention to the features of lettuce, and the texture features of lettuce are clearer than the original network. This indicates that the introduction of the ECA module can effectively improve the learning ability of lettuce features in the network. When using the BiFPN structure, it can be seen that the receptive field of the feature map is enlarged, and the focus of the model is still on the lettuce part in the red area of the feature map. This reflects the effect of BiFPN structure on adjustable weight learning for different features. After replacing the traditional convolution and C3 modules with GSConv and VoVGSCSP modules in the neck, the output layer has a more accurate localization of defect leaf features. This indicates that GSConv performs better than traditional convolution. The above experimental results demonstrate the effectiveness of the three improvement strategies in this study.

**Figure 12:**
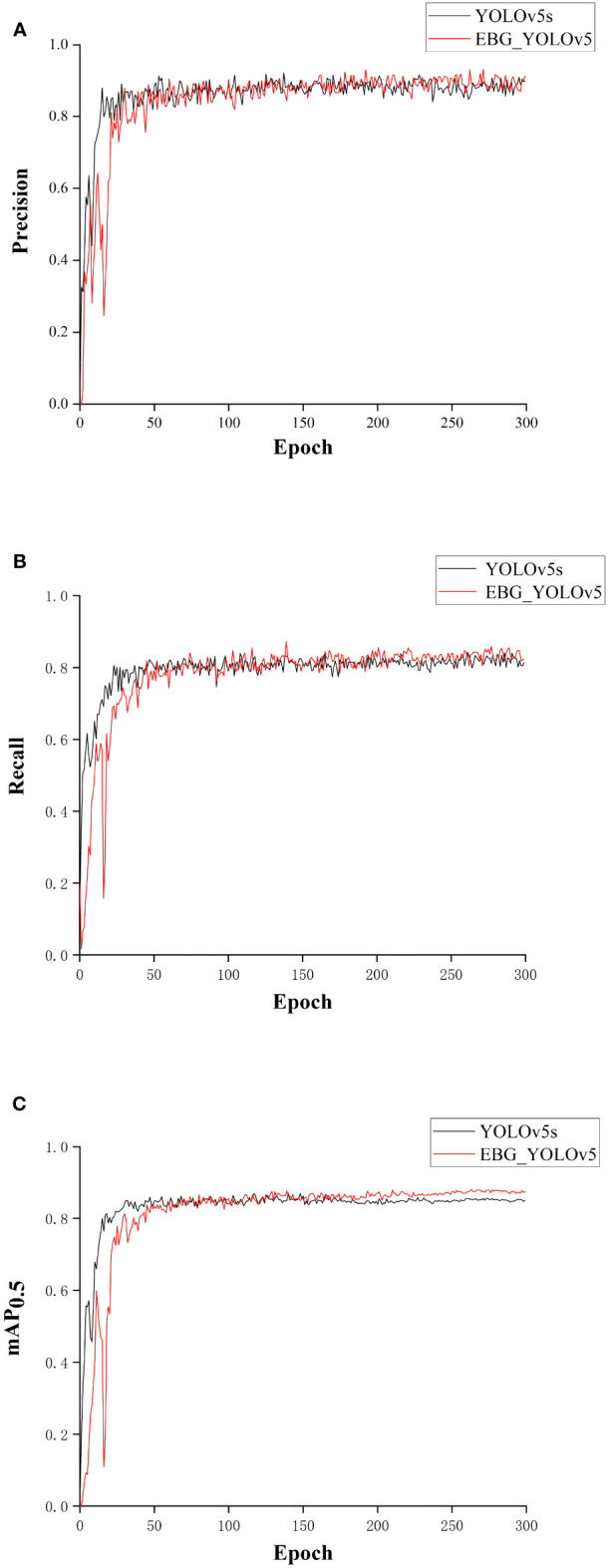
Comparison of result between EBG_YOLOv5 and YOLOv5s: **(A)** Comparison of Precision between EBG_YOLOv5 and YOLOv5s; **(B)** Comparison of Recall rate between EBG_YOLOv5 and YOLOv5s; **(C)** Comparison of mAP_0.5_ between EBG_YOLOv5 and YOLOv5s.

The Precision, Recall rate and mAP_0.5_ comparison curves between the original YOLOv5s and the EBG_YOLOv5 are shown in **Figure 12**. It can be seen intuitively from **Figure 12** that the convergence rate of EBG_YOLOv5 is inferior to that of YOLOv5s. However, the Precision, Recall rate, and mAP_0.5_ of EBG_YOLOv5 have demonstrated improvement.

**Table 3** presents the comparison of mean accuracy between YOLOv5s and EBG_YOLOv5 for each class. As can be seen from **Table 3**, the EBG_YOLOv5 has improved the recognition accuracy of four types of defective leaves. The recognition accuracy for decayed leaves was improved by 3.3%, broken leaves by 7.7%, yellow leaves by 0.6%, and wilted leaves by 1.1%.

**Table 3:** Comparison of all class accuracy between the EBG_YOLOv5 and YOLOv5s.

Category	YOLOv5s	EBG_YOLOv5
D (Decayed)	77.8	81.1
B(Broken)	74.6	82.3
Y(Yellow)	90.0	90.6
W(Wilting)	98.0	99.1

### 4.3 Comparison experiment of different algorithm model

In order to compare the effectiveness and performance of the EBG_YOLOv5 and other models, we selected SSD, Faster-RCNN, YOLOv3, YOLOv4, YOLOv7 (Wang et al., 2023) and YOLOv5m for comparative experiments under the same experimental environment and parameters.

**Figure 13:**
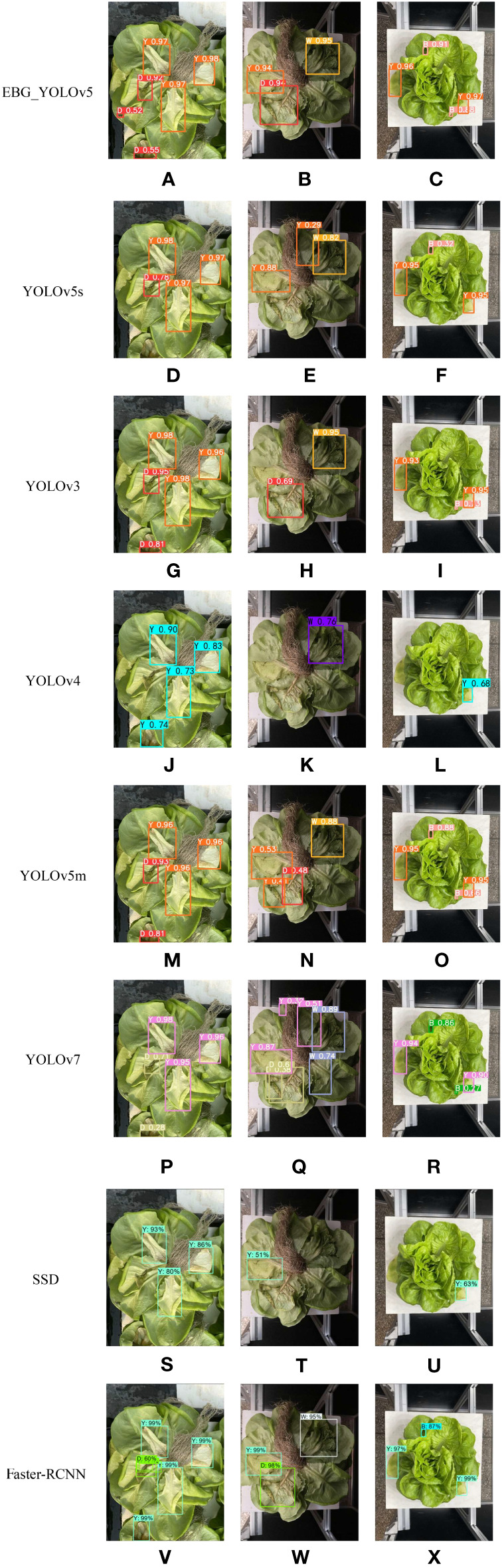
Test results for different algorithms. **(A–C)** EBG_YOLOv5 testing effect. **(D–F)** YOLOv5 testing effect. **(G–I)** YOLOv3 testing effect. **(J–L)** YOLOv4 testing effect. **(M–O)** YOLOv5m testing effect. **(P–R)** YOLOv7 testing effect. **(S–U)** SSD testing effect. **(V–X)** Faster-RCNN testing effect.

**Figure 13** shows the comparison of the test results for EBG_YOLOv5, YOLOv5s, YOLOv3, YOLOv4, YOLOv5m, YOLOv7, SSD and Faster R-CNN. It can be seen from the comparison of **Figure 13A** with D, G, J, M, and P that EBG_YOLOv5 can effectively detect the defective leaves at the edge of the image; It can be seen from the comparison between **Figures 13B** and E, H, K, N, Q, T and W that the detection effects of the other six models are affected by the change of environmental brightness, resulting in missed detection and false detection, while EBG_YOLOv5 can still accurately detect the defective leaves in the image; As can be seen from the comparison of **Figure 13C** with F, I, L, O, R, U, and X, EBG_YOLOv5 can detect small target defects in the image with higher confidence.

From **Table 4**, it can be seen that compared to other models, the EBG model proposed in this study has smaller weights. In terms of detection accuracy, EBG_ YOLOv5 is better than YOLOv5s and also better than the YOLOv5m model. Compared to the other five models, EBG_ YOLOv5 shows better performance. In terms of detection speed, EBG_ YOLOv5 and YOLOv5 are basically the same. Compared to the other six detection algorithms, EBG_ YOLOv5 has higher FPS. Therefore, in contrast, EBG_ YOLOv5 has advantages in detection performance.

**Table 4:** Comparison of experimental results between various models.

Model	mAP_0.5_/%	Weights/MB	Detection Speed (FPS)
SSD	74.5	103	41.1
Faster-RCNN	82.8	315	11.0
YOLOv3	84.3	235	46.2
YOLOv4	84.6	244	48.6
**YOLOv5s**	**85.4**	**13.7**	**60.9**
YOLOv5m	86.0	40.2	46.5
YOLOv7	84.9	71.3	51.8
**EBG_YOLOv5**	**88.0**	**11.6**	**61.7**

The bold values represent the original and improved models.

## 5 Conclusion and discussion

We have studied recent research on defect detection in lettuce and compiled it into a table, as shown in **Table 5**. The dataset in the literature mainly consists of a single object in a laboratory environment and multiple objects in a field or greenhouse environment. The main detection objects are defective leaves, diseases, and lettuce seedlings. From **Table 5**, it can be seen that the optimized network is superior to the original network, indicating that improving the network is effective.

**Table 5:** Some researches on lettuce detection in recent years.

Object	Dataset condition		Network	Improve	mAP /%	Re /%
	Defect condition	Environment condition				
Tip-Burn on Lettuce. (Munirah Hayati Hamidon and Tofael Ahamed) (Hamidon and Ahamed, 2022)	Tip-Burn	Plant factory	CenterNet YOLOv4 YOLOv5	N	78.1 67.6 82.8	58 74 79.4
Abnormal hydroponic lettuce. (Wu, Yang, wang, et al.) (Wu et al., 2022)	Yellow leaves, withered leaves, and decayed leaves	Laboratory	DeepLabV3	Y	83.26	
Lettuce disease. (R. Abbasi, P. Martinez, and R. Ahmad)	Lettuce-DownyMildew and Lettuce-Bacterial Leaf Spot	Greenhouse	YOLOv5 Faster-RCNN	N	82.13 76.34	
Hydroponic lettuce seedlings status. (Li, Yang, Guo and Yue)		Greenhouse	Faster-RCNN	Y	86.2	89.85
Hydroponic lettuce defective leaves. (this paper)	Decayed leaves, broken leaves, yellow leaves, and withered leaves.	Greenhouse and Laboratory	YOLOv5	Y	88.0	84.3

Intelligent detection of hydroponic lettuce defective leaves after harvesting is of great significance to hydroponic lettuce quality and value assurance. This study proposed a method for detection of defective leaves of hydroponic lettuce based on improved YOLOv5. The ECA module was integrated into the backbone of YOLOv5 to enhance detection accuracy. Then, BiFPN pyramid structure was introduced to enhance feature fusion and improve the retention rate of each type of feature information in the model. Finally, the GSConv and VoVGSCSP module were incorporated into the Neck part. This not only enhances model accuracy, but also reduce parameters and calculations.

The ablation experiments showed that in comparison to the YOLOv5s model, the proposed EBG_YOLOv5 have a rise in Precision, Recall rate, and mAP_0.5_ by 0.1%, 2.0%, and 2.6%, respectively, while the weights, GFLPOs and parameters decreased by 15.3%,18.9% and 16.3%. The comparison experimental results proved that the proposed EBG_YOLOv5 model enhances the accuracy in detecting defective leaves of hydroponic lettuce and optimizes the identification of small target leaves and root occlusion. It achieves higher performance with a smaller memory footprint than other mainstream target detection models.

The establishment of defect leaves identification model of hydroponic lettuce can provide technical support for related quality detection equipment. In future work, we will increase the variety of lettuce in the dataset to further improve the applicability of the model and continue to optimize the model to prepare for the subsequent research on the quality detection and grading equipment of hydroponic lettuce.

## Data availability statement

The raw data supporting the conclusions of this article will be made available by the authors, without undue reservation.

## Author contributions

XJ: Conceptualization, Supervision, Formal analysis, Project administration. HJ: Methodology, Software, Writing-original draft, Writing-review and editing. CZ: Validation, Visualization. ML: Investigation, Formal analysis. BZ: Data curation. GL: Project administration. JJ: Supervision. All authors contributed to the article and approved the submitted version.
